# A mutation in the type II hair keratin *KRT86* gene in a Han family with monilethrix^☆^

**DOI:** 10.1016/S1674-8301(11)60006-7

**Published:** 2011-01

**Authors:** Jin Wu, Yongli Lin, Wenrong Xu, Zhongming Li, Weixin Fan

**Affiliations:** Department of Dermatology, the First Affiliated Hospital of Nanjing Medical University, Nanjing, Jiangsu 210029, China

**Keywords:** hair diseases, keratin, monilethrix, hHB6, mutation

## Abstract

Monilethrix, a congenital disease of hair, is usually associated with mutations in keratin genes, like *KRT81*, *KRT83* and *KRT86*. We conducted this study to investigate the mutation of type II human basic hair keratin *hHb/KRT* gene in a Han family with monilethrix and obtain information for potential pathogenic mechanism study of monilethrix. Peripheral blood samples were drawn for genomic DNA detection. Exon 1 and exon 7 of the *KRT81*, *KRT83* and *KRT86* genes were amplified by PCR. All PCR products were sequenced directly using an ABI 310 DNA sequencer. These sequences were aligned with the standard sequences in GenBank using the BLAST software. PCR products were digested with restriction endonuclease and restriction fragment length polymorphism (RFLP) analysis was performed. In this study, we identified one novel mutation, which is a heterozygous transitional mutation of G→A at position 1,289 in exon 7 of the *KRT86* gene [R430Q (*KRT86*)]. RFLP assays for the novel mutation excluded the possibility of polymorphism. The R430Q mutation of the *KRT86* gene may be pathogenic for monilethrix. Meanwhile, we did not find any novel mutation or recurrent mutation in exons 1 and 7 of *KRT81* and *KRT83* and exon 1 of *KRT86*. There is a potential pathogenic gene in the subjects and our results expand the spectrum of mutations in the *hHb6* gene.

## INTRODUCTION

Monilethrix is a congenital defect of hair, with a high penetrance and variable expression[1],[2]. The disease was first reported by Smith in 1829[3]. Affected individuals may have normal hair at birth, but within the first few months of life, they develop fragile, brittle hair, which then tends to fracture and produce varying degrees of dystrophic alopecia. In mild cases, only the occipital region of the scalp is involved; however, in severe cases, the secondary sexual hair, the eyebrows, and the eyelashes may also be involved[4]. Follicular hyperkeratosis has a tendency to occur on the scalp, the nape of the neck and the extensor surfaces of the upper arm and thigh. Light microscopic and scanning electron microscopic (SEM) examination can distinguish and display elliptical nodes of normal thickness and intermittent narrowing and internodes. The hair is easily disrupted at internodes. Ultrastructurally, vacuolation and alterations in the fibrillar structures of lower cortex cells have been described[5]. Occasionally, regrowth of apparently normal hair may occur during puberty or pregnancy, but the lesion never disappears completely[6].

Generally, monilethrix is inherited in an autosomal dominant fashion and associated with mutations in three keratin genes (*KRT81*, *KRT83*, and *KRT86*) located on chromosome 12q13[7]–[10]. In this study, we have identified a Chinese family with monilethrix, and sequenced three keratin genes (*KRT81*, *KRT83*, and *KRT86*) of the subjects to elucidate the underlying molecular basis of this disorder.

## MATERIALS AND METHODS

### Clinical features of patients

**Fig.1:**
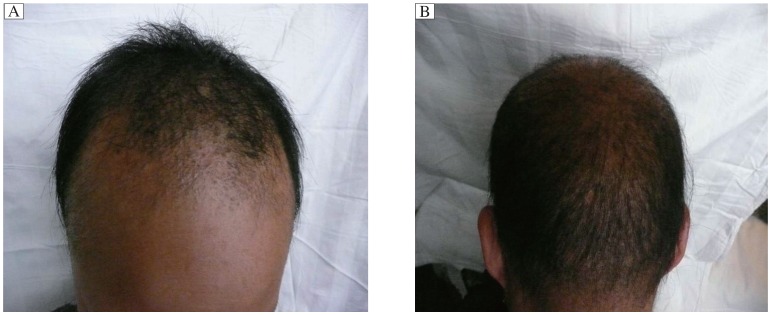
Scalp hair dystrophy in two monilethrix patients. A: The hair status of the proband (III-3) patient. The hair shaft showed nodes at regular intervals of 0.5-0.7 mm and was thin and brittle. Hair loss was concentrated in the forehead region and both temples, which was exacerbated by mechanical stress such as wearing a cap. Hairs would fall off at their ends. Hairs in general were short and brittle, but some hairs reached a length of up to 20 cm. There was pronounced follicular hyperkeratosis on the forehead. B: The hair picture of the patient's father (II-3), whose hairs were also sparse and fragile. The scalp hairs were short, dry, brittle and lusterless, and most of them emerged from keratotic follicular papules and the hairs broke easily, but the eyebrows were normal.

**Fig. 2:**
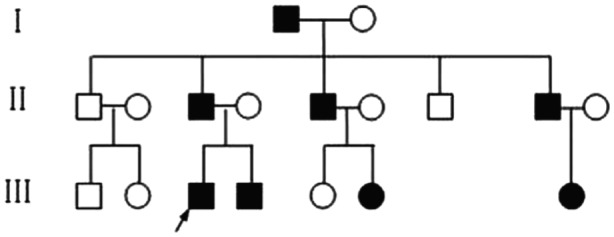
Summary of the family tree. The proband patient was indicated by the arrow.

The proband (III-3) was an 8-year-old boy. He visited our department with a complaint of increasing hair loss and inability to grow long hair. At birth, the hair shaft appeared normal but soon thereafter, nodes began to form along the shafts at regular intervals of 0.5-0.7 mm and the hair grew slowly, and became thin and brittle. The hair loss was concentrated in the forehead and both temples, and was exacerbated by mechanical stress such as cap wearing. Hairs could fall off at the end. In general, hairs were short and brittle, but some reached a length of up to 20 cm. There were no other subjective symptoms. Upon examination, we found that the hairs were sparse and withered, with a pronounced follicular hyperkeratosis in the forehead region (***Fig. 1A***) and also on the neck and the extensor aspect of four limbs. The eyebrows and eyelashes were uncompleted (not shown). His father (II-3), who was 40 years, was also affected, with sparse and fragile hair since birth. His scalp hairs were short, dry, brittle, lusterless and were broken easily, and most of them emerged from keratotic follicular papules. His eyebrows were normal but axillary hairs were absent and pubic hairs were sparse. His nails and teeth were normal and had no other complications. He never received any treatment. In middle age, his hair symptoms gradually improved. Long terminal hairs increasingly appeared, but short and thin hairs and perifollicular papules also remained (***Fig. 1B***). His symptoms were reported to be serious in winter but mild in summer. To be noted, one uncle (II-7) of the proband had normal hairs but loss in hearing and visual senses. The pedigree of the three-generation monilethrix family is shown in ***Fig. 2***.

### Scanning electron microscopy

Hair samples from the patients were examined by SEM (PHILIPS XL30 ESEM TMP), which was performed at Nanjing Agricultural University.

### Source of DNA

The study was approved by the Ethics Committee of Nanjing Medical University and informed consent was obtained from all the study participants. Peripheral blood samples were obtained from the 8 affected patients and 10 unaffected members of this family and from 100 unrelated healthy Chinese individuals. Genomic DNA was extracted using a puregene kit (RN0601/02) from Galen BeiJin Inc., China.

### Analysis of the *KRT81, KRT83* and *KRT86* gene

The exons of the *KRT81,* *KRT83* and *KRT86* gene along with the adjacent sequences of the exon–intron borders were amplified by PCR. The sequences of the primers for the *KRT81*, *KRT83* and *KRT86* genes were reported previously[5], and are listed in ***Table 1***. PCR was performed using Advantage^™^ 2 DNA polymerase (Clontech, Japan).

**Table 1:** The primer sequences for KRT81, KRT83 and KKT86

Genes	Exon No.	Forward primer (5′-3′)	Reverse primer(5′-3′)
*KRT81*	Exon1	TCACAGCCAAGCCCCTTCAG	GGCAGGCAGAGGTCTTTGTG
	exon7	GCTTGGTGGGGAGTGTGGTC	TCAGCCAAGGCCAAGGGTAG
*KRT83*	Exon1	TCTTTGCTCCCTCTTTTAACACCAG	TCAGTGCCCAGCCTGAGTTC
	exon7	CTATTGGAGACTGGCTGGGATTTC	GATCCATAGAGGCAAGAATGTCACC
*KRT86*	Exon1	AAAAGGCCTACAGAGGTGCAAG	GGCCCTGGCTGTGTAGGTG
	exon7	AGCTTGGTGGGGAGCATGG	AATGCTGCCAGGAGTGTGAGG

After an initial denaturation of 3 min at 96°C, 30 cycles were performed (96°C for 30 s, 56°C for 45 s, and 72°C for 30 s), followed by a final extension of 7 min at 72°C. The amplified PCR fragments were analyzed on 1.5% agarose gels. After gel extraction of the fragments, direct fluorescent chain-termination DNA cycle sequencing was performed (Big Dye DNA Sequencing kit, Applied Biosystems, USA). The DNA sequences were analyzed on an ABI 310 DNA sequencer (Applied Biosystems). The mutations were identified by visual inspection and comparison with control sequences from unrelated, unaffected individuals.

## RESULTS

### Light microscopy and SEM observation

**Fig. 3:**
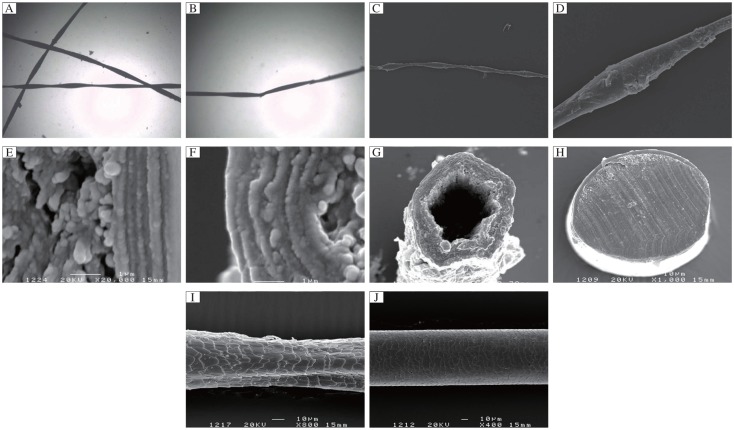
Light microscopy and SEM observation. Light microscopic examination of plucked hairs showed typical features of monilethrix. The beading could be gross or subtle. The breaks in the hair shafts always occurred at internodes (A, B). SEM revealed that the thickness of the hair shaft was slightly, but obviously, inconsistent in some parts, resulting in nodes and internodes. The nodes seemed to be of normal thickness, but the internodes were abnormally thin (C, D). The internodes did not show a constant periodicity. The formation of fissure or cavities were evident in the transverse section of the hair shafts of the patients (E, F). This phenomenon was more serious in the transverse section of the hair shafts of the grandfather (E) than that of the proband (F). G shows that the transverse sections of the hair shafts lack a medulla. The normal transverse sections of the hair shafts had a cortex and medulla and the transverse section was well distributed (H). The hair cuticle exhibited a jagged shape and were fragmentated or lost (I) compared to the hair cuticle in a normal person, which was slick and well arranged (J).

Light microscopic examination of plucked hairs showed typical monilethrix of the patients. The beading could appear gross subtle, and the breaks in the hair shafts always occurred at internodes (***Fig. 3A*** and ***Fig. 3B***). SEM revealed that the thickness of the hair shaft was slightly, but obviously, inconsistent in some parts, resulting in nodes and internodes. The nodes seemed to be of normal thickness, but the internodes were abnormally thin (***Fig. 3C*** and ***Fig. 3D***), and the latter did not show a constant periodicity. The formation of fissure or cavities were evident in the transverse section of the hair shafts of the patients (***Fig. 3E*** and ***Fig. 3F***). This phenomenon was more serious in the transverse section of the hair shafts of the grandfather (***Fig. 3E***) than that of the proband (***Fig. 3F***). ***Fig. 3G*** shows the transverse sections of the hair shafts lacking a medulla. The normal transverse sections of the hair shafts had a cortex and medulla, and were well distributed (***Fig. 3H***). The hair cuticle exhibited a jagged shape and was fragmentated or lost (***Fig. 3I***) compared to the hair cuticle in a normal person, which was slick and well arranged (***Fig. 3J***).

### Identification of a new mutation in the *KRT86* gene

**Fig. 4:**
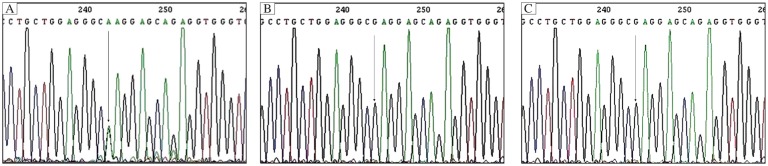
Mutation in the *KRT86* gene. A: The mutation in all patients is a G-to-A transition at nucleotide position 1,289 in exon 7, and the mutation occurs in the second position of an arginine residue (CGA to CAA), resulting in the substitution of arginine at amino acid 267 by glutamine (R430Q). The mutation R430Q was not identified in 10 unaffected controls (B) and 100 normal controls (C).

The light microscopy and SEM findings suggested that our patients represented lesions of monilethrix. We then analyzed the genomic DNA for type II hair keratin *KRT81*, *KRT83*, and *KRT86* genes. Mutation analysis was carried out of the gene sequences coding for the helix initiation motifs (HIMs) and helix termination motifs (HTMs) of the three type II hair keratins in which mutations associated with monilethrix have been reported. Direct sequencing of the PCR-amplified gene regions encoding the HIMs of the various keratins did not reveal deviations from the reported sequences in both affected and unaffected members of the family. However, analyses of the HTM-encoding regions of the *KRT86*, *KRT83* and *KRT81* gene led to the detection of a heterozygous G to A point mutation in the *KRT86* gene in the family (***Fig. 4A***). The *KRT86* gene mutation was found in all eight affected family members with clinical manifestations (***Fig. 2***). The mutation identified in all patients was a G-to-A transition mutation at nucleotide 1,289 in exon 7, (CGA to CAA), resulting in the substitution of arginine at amino acid 267 by glutamine (R430Q) (***Fig. 4A***). The mutation R430Q was not found in 10 unaffected members of this family (***Fig. 4B***) and 100 unrelated healthy Chinese individuals (***Fig. 4C***). The results showed that the patients had the R430Q mutation heterogeneously, whereas 10 unaffected members of this family and 100 control individuals were homozygotes for the wild-type sequence. We found that there were no novel mutations or recurrent mutations in the entire sequence of exons 1 and 7 of *KRT81* and *KRT83* and exon 1 of *KRT86* among the patients.

### Mutations in the *KRT86* gene confirmed by RFLP analysis

**Fig. 5:**
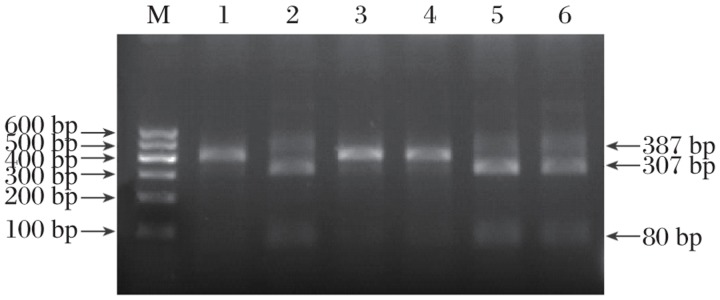
RFLP analysis. Wildtype 387-bp fragments that containing *Bse*R1 restriction site were completely digested into 80- and 307-bp fragments in normal control samples (lanes 2, 5 and 6). On the other hand, the mutant allele lacked the *Bse*R1 restriction site and was not digested by the enzyme in the patients (lanes 1, 3 and 4).

In order to confirm this mutation, the PCR products were digested with *Bse*R1 and resolved by agarosegel electrophoresis. As shown in ***Fig. 5***, the wildtype 387-bp fragments containing a *Bse*R1 restriction site were completely cleaved into an 80- and a 307-bp fragment in unaffected members of this family (lane 2 in ***Fig. 5***)and in normal control (lane 5 and 6 in ***Fig. 5***). On the other hand, the mutant allele lacked the *Bse*R1 restriction site and was not digested by the enzyme (lane 1, 3 and 4 in ***Fig. 5***). These findings confirmed the presence of a G→A substitution in exon 7 of *KRT86* in the patients.

## DISCUSSION

Monilethrix is a rare developmental disease that is characterized by small elliptical node-like deformities with increased hair fragility, resulting in partial or diffuse alopecia. This disorder has been reported to be caused by mutations in the HTMs or HIMs of three type II cortex keratin genes (*KRT81*, *KRT83* and *KRT86*). In this study, we described a novel Arg→Gln mutation caused by a G→A point mutation in the second position of the CGA codon of Arg430 in the type II cortex *KRT86* gene of a monilethrix patient in a Chinese family of Han ethnicity. The same type of mutation has not been found previously in the type II cortex keratin *KRT86* gene of affected members. To our knowledge, because of the linkage of monilethrix to the type II keratin locus[11],[12], the gene regions coding for the HIM and HTM of type II hair keratins have been analyzed for possible mutations. In different families, different types of mutations in the HTMs of hair keratin *KRT86*, as well as an HTM E413K mutation in hair keratin *KRT81*, have been reported[8],[13]. A *KRT83* mutation was reported in a single monilethrix family in 2005[10]. The reason for the low mutation rate in *KRT83* is unknown. Among the *KRT86* mutations, the non-conservative E413K substitution was by far the most frequent mutation, followed by the conservative E413D and the non-conservative E402K substitutions[14]. However, we have not found these frequent mutations in *KRT86* and the less frequent mutation in *KRT83* in our patients.

Most mutations described to date have been located in the *KRT81*, *KRT83* or *KRT86* gene and are subject to speculation. ***Table 2*** summarizes the mutations reported in monilethrix, including most of the data of monilethrix in the world and all the data of the mutations of monilethrix in China. ***Table 2*** lists mutations associated with monilethrix including 9 types of mutations in *KRT86* and 1 type of mutation in *KRT83*, as well as two kinds of mutations in *KRT81*. Among these, 2 out of 9 mutations at codon 413 in *KRT86* and 1 of 2 mutations at codon 413 in *KRT81* have been reported. Thus, codon 413 of *KRT86* and *KRT81* is thought to be a possible mutation hot spot in monilethrix. Most mutations reported show amino acid substitutions at the end of the 2B domain in the HTM of both hair keratin genes. These two mutations, E402K and E413K in *KRT86*, have been previously documented in monilethrix patients. The number of patients with E413K in *KRT86* is greater than that of patients with other mutations. This outcome implies that this site, the HTM of *KRT86*, may be a ‘hot spot’ for mutagenesis in monilethrix. However, these two recurrent mutations, E402K and E413K, are the most common mutations known in monilethrix. In the present study, this tendency was not confirmed in the monilethrix family. Until now, pathogenic mutations in monilethrix seem to have been restricted to type II cortex keratins.

**Table 2:** Pathogenetic mutations in three hair keratin genes *KR81, KR83* and *KR86*

Nucleotide Change	Amino acid change	Position	Origin of genecology
KRT86			
A→G	N114D	HIM	Sweden[15], Portugal[16]
A→C	N114H	HIM	Scotland[16]
C→A	A118E	HIM	France[17]
G→C	E402Q	HTM	Wales[18]
G→A	E402K	HTM	USA[15], Greece[18], Israel[9], Australia[19] China[20]–[23]
G→T	E413D	HTM	France[26], Israel[9],[24]
G→A	E413K	HTM	UK[13],[16],[25], Germany[8],[9],[18], Israel[8],[9] Japanese[27], Indian[28]
G→A	R430Q	HTM	This paper
KRT83			
G→A	E407K	HTM	Dutch[10]
KRT81			
G→A	E402K	HTM	France[28], Ireland[16], Germany[16]
G→A	E413K	HTM	Canada[8], Scotland[16], Italy[19]

HIM: helix initiation motif; HTM: helix termination motif.

In this family, we identified a glutamine substitution of the corresponding arginine acid residue, Arg430, in the type II hair keratin *KRT86* gene in all eight affected individuals, suggesting that this site represents a new mutational hot spot in these highly related type II hair keratins. The *KRT86* gene is mainly expressed in cortical trichocytes of the hair shaft. This indicates that monilethrix is a disease of the hair cortex. Arginine is a basic amino acid that carries a positive charge; however, glutamine is a neutral amino acid that does not carry a charge. The change in charge may result in an abnormality of the formation of the intermediate filament proteins, causing a defect in the hair keratins.

On the other hand, Oshimura *et al.*[29] measured the protective effects of arginine in an oxidative coloring or bleaching process through the methods of contact angle measurement, tensile measurement and amino acid analysis. Their results suggested that arginine prevents an undesirable attack by hydrogen peroxide on hair proteins and hair surface lipids. Furthermore, it has also been suggested from amino acid analysis that a considerable amount of arginine is deposited on or in hair fibers with coloring agents.

The variable clinical expressions of monilethrix is evident. Previous studies have indicated that there is no clear correlation between the phenotype and genotype of monilethrix and the clinical phenomenon varies among patients. The clinical phenomenon of the mutant gene may involve serious alopecia and also hair follicle keratinization, even with only carrier status. In our study, intrafamilial variation was evident. Family members had mild to moderate scalp involvement with moniliform hairs. In the mildest form, the disease involved only the occiput and nape of the neck, but in other family members with severe forms, most areas of the scalp, eyebrows and hairs on the legs were also involved. The differences in clinical phenomena also support the conclusion that a correlation between the phenotype and genotype of monilethrix has not been established.

This study emphasizes the key role of hair keratin K86 in the pathogenesis of monilethrix, which also demonstrates the heterogeneity of this disease and its potential for new mutations.
